# Epigenetic Regulation by lncRNAs: An Overview Focused on UCA1 in Colorectal Cancer

**DOI:** 10.3390/cancers10110440

**Published:** 2018-11-14

**Authors:** Bernadette Neve, Nicolas Jonckheere, Audrey Vincent, Isabelle Van Seuningen

**Affiliations:** Inserm UMR-S 1172, Centre de Recherche Jean-Pierre AUBERT Neurosciences et Cancer (JPArc), Team “Mucins, Epithelial Differentiation and Carcinogenesis”; University Lille; CHU Lille, 59045, Lille CEDEX, France; nicolas.jonckheere@inserm.fr (N.J.); audrey.vincent@inserm.fr (A.V.); isabelle.vanseuningen@inserm.fr (I.V.S.)

**Keywords:** long non-coding RNA (lncRNA), lncRNA Urothelial Cancer Associated 1 (UCA1), colorectal cancer (CRC), competing endogenous RNAs (ceRNA)

## Abstract

Colorectal cancers have become the second leading cause of cancer-related deaths. In particular, acquired chemoresistance and metastatic lesions occurring in colorectal cancer are a major challenge for chemotherapy treatment. Accumulating evidence shows that long non-coding (lncRNAs) are involved in the initiation, progression, and metastasis of cancer. We here discuss the epigenetic mechanisms through which lncRNAs regulate gene expression in cancer cells. In the second part of this review, we focus on the role of lncRNA Urothelial Cancer Associated 1 (UCA1) to integrate research in different types of cancer in order to decipher its putative function and mechanism of regulation in colorectal cancer cells. UCA1 is highly expressed in cancer cells and mediates transcriptional regulation on an epigenetic level through the interaction with chromatin modifiers, by direct regulation via chromatin looping and/or by sponging the action of a diversity of miRNAs. Furthermore, we discuss the role of UCA1 in the regulation of cell cycle progression and its relation to chemoresistance in colorectal cancer cells.

## 1. Colorectal Cancer

Colon and rectal cancers (together nominated colorectal cancer (CRC)) have become the second leading cause of cancer deaths both in the United States and in Europe ([1,2], respectively). CRC occurrence has been correlated to an unhealthy lifestyle (tobacco, alcohol, red meat, sedentariness, obesity), whereas physical activity and dietary fibers protect against CRC [3]. In addition, early diagnosis by stool-based CRC screening has decreased disease mortality [4]. However, most patients are only diagnosed after they have symptoms and frequently present metastatic lesions (e.g., 14% in the German DACHS study [5], 19–24% in US SEER study [6]). An additional 20% of the CRC patients develop metastases during their disease evolution [5,7]. Distant metastases occur mainly in the liver, peritoneum and lung tissues. Non-metastatic colon cancer is generally treated by surgical colectomy combined with chemotherapy (e.g., inhibitors of DNA synthesis, FOLFOX (FOLinic acid, 5-Fluorouracil (5-FU) and OXaliplatin) or CAPEOX (CAPEcitabine and OXaliplatin), ±anti-VEGF antibodies such as bevacizumab [8]). Isolated and local metastases of colon cancer are also surgically resected. For unresectable metastatic CRC, a continuum of systemic chemotherapy is provided (e.g., combined therapy with reduced folate leucovorin, topoisomerase I inhibitor irinotecan, anti-EGFR antibodies cetuximab, or panitumumab; described in the National Comprehensive Cancer Network Clinical Practice Guidelines [8]).

Several tumor tissue alterations are important for the diagnosis and prognosis of CRC [8,9]. For example, CRC patients present 5–20% tumors with microsatellite instability (MSI) related to mismatch repair (MMR) defaults, while 15% of the tumors have a CpG island methylation phenotype. CRC patients with a primary right-sided tumor have significantly greater rates of MSI and may have no benefit of 5FU chemotherapy at stage II of CRC [10]. In addition, the tumor tissue of metastatic CRC patients may present RAS (KRAS and NRAS) or B-RAF mutations that result in a constitutively active MAPK signaling pathway, which increases cellular proliferation. KRAS mutated tumors lack a response to EGFR inhibitors (e.g., cetuximab), whereas inhibitors of mutated B-RAF (e.g., vemurafenib) may be used in combination with chemotherapy [8]. In addition, classifying tumors according to gene expression-based molecular subtypes prognoses the response to therapy, and may innovate towards personalized therapy [11,12]. Actually, colon and rectal cancers are frequently analyzed in epidemiological studies as one entity, although differences in clinical and molecular characteristics of primary colon cancers were recently re-highlighted between tumors from the right side, including the caecum, ascending colon, hepatic flexure and two-thirds of the transverse colon (~27% of patients), and one from the left-sided colon, including the distal third of the transverse colon, splenic flexure, descending colon, sigmoid and rectum [13,14,15]. In these studies, the tumor location was found to be an independent prognostic factor for overall survival, which may be worse for patients with right-sided tumors [13]. The reported 5-year survival rate for patients with non-metastatic CRC is 70–90%, whereas for patients with metastatic CRC this was a poor 14% [16]. This bad prognosis could be due to the acquired cellular chemoresistance and the presence of residual colon cancer cells.

## 2. Mechanisms of Regulation by Long Non-Coding RNAs in Cancer Cells

**Figure 1:**
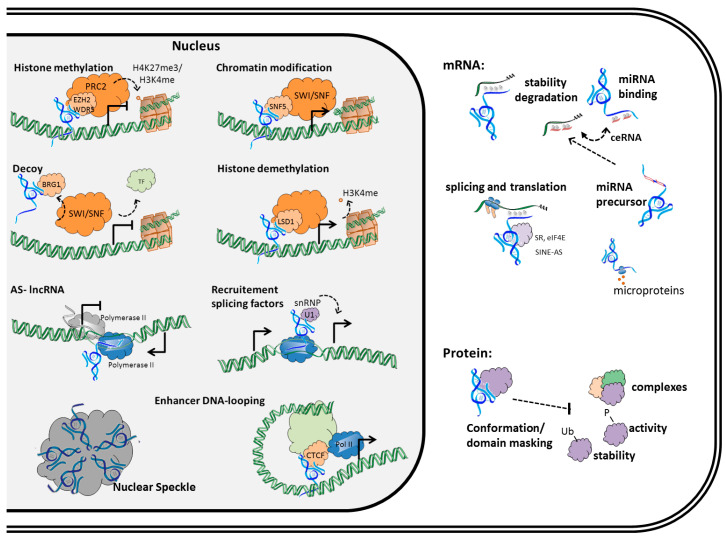
The function of lncRNAs in the cell. LncRNAs exert different functions in the nucleus, ranging from genomic DNA organization in speckle bodies, histone, and DNA methylation and direct transcriptional regulation (Section 2.1). Through their interaction with mRNA and function in miRNA regulation, they affect protein translation (Section 2.2). In addition, they interact with proteins, affecting stability, activity, and/or complex recruitment (Section 2.3).

Long non-coding RNAs (lncRNAs) are a heterogeneous class of RNAs that are arbitrarily defined as transcripts over 200 nucleotides long and lacking sequences encoding functional and/or conserved proteins. According to the current human GENCODE Release, 27% of all genes are lncRNA transcripts (15,779 transcripts, Release v28, https://www.gencodegenes.org). These lncRNAs are implicated in a diversity of physiological processes and a large range is implicated in CRC [17,18,19]. Moreover, differential lncRNA expression was related to different clinical CRC characteristics and molecular phenotypes [19,20]. LncRNA-mediated regulation has crucial roles in gene expression control, which implicates mechanisms based on both base-pair interactions (DNA/RNA) and protein interactions (Figure 1).

### 2.1. Interaction of lncRNAs with DNA

The lncRNAs-DNA interactions affect both DNA organization and transcription.

#### 2.1.1. DNA Organization 

Genomic DNA is packed in the nucleus into a higher order genome organization, which has a dynamic and spatial architecture. Within the nucleus, the nucleoli and paraspeckles display a unique morphology, positioning, and are related to transcriptional activity (review by References [21,22]). The lncRNAs, NEAT1 and MALAT1, were shown to play a role in the forming and organization of these nuclear speckle bodies. Both these lncRNAs are related to active chromatin sites in the nucleus, are overexpressed in CRC, and are correlated with a poor disease prognosis (References [23,24,25,26,27], respectively). 

The packaging of genomic DNA also depends on histone and DNA modifications, which are regulated by epigenetic complexes, and that may bind lncRNAs. A common feature is the potential of lncRNAs to interact with the polycomb repressive complex-2 (PRC2), in which the principal subunits are subunit SUZ12, embryonic ectoderm development (EED) and the enhancer of zeste homolog 2 (EZH2). RNA-Immunoprecipitation experiments with SUZ12 and EZH2 showed that 20–30% of the intergenic lncRNAs interact with PRC2 [28]. In CRC cells, EZH2 has been shown to bind to at least 12 lncRNAs, including UCA1 (Table 1). LncRNAs recruit PRC2 towards gene promoters/enhancers and thus stimulate epigenetic silencing by trimethylation lysine 27 of histone H3 (H3K27me3) (reviewed by Reference [29]). Interestingly, the tumor suppressor KLF2 gene is reported to be silenced in CRC cells by several lncRNAs through this mechanism (e.g., SH3PXD2A-AS1, HOXA-AS2 [30,31]), whereas for most genes, only a unique lncRNA/EZH2 combination is known. In addition, other CRC-related lncRNAs interact with adaptor protein WDR5 from the histone H3 lysine 4 (H3K4) methyltransferase-complex (Table 1). Inversely, lncRNAs may also affect histone demethylation. The interaction with histone lysine-specific demethylase 1 (LSD1) was shown for four different CRC-associated lncRNAs (Table 1). DNA methylation is affected by lncRNAs through several mechanisms. On the one hand, expression of DNA-methyl transferases is frequently regulated by lncRNAs through interference with miRNA-mediated transcript decay. On the other hand, some studies showed a physical interaction with DNMTs, including NEAT1 [32]. LncRNAs also affect chromatin configuration and DNA methylation via interaction with switching defective/sucrose non-fermenting (SWI/SNF) complexes (Reference [33]). LncRNAs, including UCA1, were shown to bind to Brahma related gene 1 (BRG1) in a variety of cancer cells (Table 1), while others bind to SNF5 and BAF200a of these SWI/SNF complexes [34,35].

#### 2.1.2. DNA Transcription 

Many lncRNAs are antisense to coding mRNA transcripts. Our text-mining using the UCSC genome table browser shows that there are at least 991 genes with a known antisense transcript (curated RefSeq track, GRCh38/hg38). The transcription of antisense lncRNA can directly inhibit transcription of sense coding genes [63,64,65], which may be mediated by Polymerase II collisions [26]. Both SPINT-AS1 and UTX-AS1 expression are negatively correlated with sense gene expression, and their overexpression is correlated with poor prognosis for CRC [66,67]. Transcripts of lncRNA may also bind to splicing machinery factors and activate proximal promoter regions, as shown for U1 snRNP/linc1319 that activated the malignant brain tumor (MBT) domain-containing protein Sfmbt2 [68]. In addition, long-range chromatin looping through lncRNA-DNA interaction may regulate protein-coding gene transcription [69,70]. Amaral et al. reported the association with chromatin looping for several lncRNAs differentially expressed in CRC cells (GAS5, H19, HAGLR, NEAT1, PINT, and CRNDE (in Supplementary Table S6 of Amaral et al. [70])). The lncRNA CCAT1-L that is upregulated in human CRC also regulates such chromatin looping at the MYC locus [71]. UCA1/CUDR promotes chromatin looping at the promoter of lncRNA HULC in liver cancer cells [72]. First reported for tumor suppressor lncRNA MEG3, one of the mechanisms that guide lncRNAs to target DNA sequences is the formation of the triplex RNA-DNA helixes of GA-rich regions [70,73]

### 2.2. Interaction of lncRNAs with RNA 

Evidence of direct physical lncRNA-mRNA interaction enhancing the stability of mRNA is sparse, but was shown for lncRNA MACC1-AS1 in gastric cancer cells and MAPT AS1 in breast cancer cells [74,75]. Similarly, the transforming growth factor-β (TGFβ)-induced antisense RNA Zeb2/Sip1 in bladder carcinoma cells binds to the 5’UTR-splicing site of the ZEB2 transcript and prevents its degradation [76,77]. Nonetheless, the stability of mRNA is modified via the binding of miRNAs frequently, which recruits a miRNA-induced silencing complex (miRISC) triggering mRNA deadenylation and decay [78]. On one hand, lncRNAs can be processed to generate miRNAs changing the miRNome of cells. Several tumor-related lncRNAs have been shown to be miRNA-precursors (Table 1). On the other hand, lncRNAs are frequently reported to function as competing endogenous RNAs (ceRNAs) to relieve the miRNA-mediated degradation of mRNAs [79]. Besides lncRNA, ceRNAs may also include mRNAs, circle RNAs, and pseudogenes [80]. Despite the fact that the stoichiometric relationship between miRNAs/regulated genes and the affinity of ceRNAs to miRNAs may question the feasibility of gene regulation through changes in ceRNA levels [81], several dedicated databases list lncRNA-miRNA interactions [80,82] and the number of original reports on their interactions still increases every year (at least 130 articles on lnc-ceRNAs January–June 2018). Recently, an lncRNA/miRNA/mRNA interactome was reported based on CRC expression data from the TCGA database. These analyses illustrated a ceRNA network of 25 principal miRNAs and 64 lncRNAs, including CRNDE, H19, HULC and UCA1 [17,83]. As discussed below, UCA1 was reported to interact with 29 miRNAs in several types of cancers. Five of these miRNAs were reported as differentially expressed and in the ceRNA network (miR-143, -144, -145, -182, and -206; [83]). It remains unclear whether the binding of miRNAs to lncRNAs also triggers lncRNA decay, since this decay may be very slow.

Direct interaction of lncRNA-mRNA affects mRNA splicing, but may also interfere with protein translation. Antisense transcripts that overlap a gene translation start site and encode an inverted retrotransposon short interspersed nuclear element (SINE) may stimulate protein translation [84]. In addition, the interaction of lncRNA GAS5 in a complex with Eukaryotic Translation Initiation Factor 4E (eIF4E) inhibited the cMYC translation [85].

### 2.3. Interaction of lncRNAs with Proteins

The interaction of lncRNAs with proteins can evoke the recruitment of effector complexes: as discussed above, the interaction with protein complexes affect epigenetic modifications and the interaction with heterogeneous nuclear ribonucleoproteins (hnRNPs) can regulate gene transcription in the nucleus (respectively reviewed by References [29,86]). The binding of lncRNAs to proteins can also function as a scaffold to increase protein stability (Table 1), e.g., the binding of the lncRNA Small Nucleolar RNA Host Gene (SNHG) 15 to SLUG blocks its ubiquitin-mediated degradation in colon cancer cells [62]. LncRNA binding to proteins can also decoy the protein function such as LINC01133 that titrates the splicing factor SRSF6 away from its targets, thereby preventing epithelial to mesenchymal transition in CRC cells [87]. Other lncRNAs also bind proteins of the splicing machinery (reviewed by Reference [88]), for example, lncRNA FAS-AS1 (SAF) interacts with SP45 resulting in alternatively spliced and anti-apoptotic FAS in cancer cells [89] and MALAT1 was shown to interact with serine/arginine (SR) splicing factors in nuclear speckle bodies [90]. Furthermore, interaction with signaling proteins may alter the activation of signaling pathways. For example, the binding of lncRNA NKILA to the NF-κB/IκB complex in breast cancer cells prevents IκB phosphorylation, thereby preventing NF-κB activation [91]. The cytoplasmic lncRNA LINK-A binding activates a protein tyrosine kinase complex (BRK/LRRK2), which results in the increased HIF1α signaling in breast cancer cells [92].

Although defined as long “non-coding” RNA, recent findings suggest that the presence of ribosomes on lncRNAs may indicate that the short open reading frames are a source of small peptide synthesis [93,94]. The detection of microproteins is challenging, but some evidence was reported; the lncRNA LINC00961 encodes a polypeptide (SPAR) that negatively regulates mTORC1 activation [95]; the LINC01420-derived microprotein was identified as a novel component of the mRNA decapping complex regulating mRNA decay [96] and a HOXB-AS3-derived peptide was shown to regulate pyruvate kinase M splicing and to affect the metabolic reprogramming of CRC cells [97]. The number of lncRNAs that are actually a source of small regulatory peptides remains to be investigated. Alternatively, the interaction of lncRNAs with ribosome complexes may trigger lncRNA degradation [98].

Accumulating evidence shows that lncRNAs are involved in the initiation, progression, and metastasis of cancer [99,100]. We have discussed how lncRNAs may regulate cancer-associated expression profiles on diverse levels. We have briefly cited several well-known lncRNAs that are implicated in CRC (Table 1), such as GAS5, H19, HOTAIR, CCAT1-L, CRNDE, and MALAT1. It has been shown that the gene desert of Chr8q24, which is a CRC risk locus, harbors 7 lncRNAs including CCAT1 (CARLo-5), and CASC19 (CARLo-6). In addition, numerous colorectal cancer associated lncRNAs, such as CCAT6 (MYCLo-2), CASC8 (CARLo-1), CASC21 (CARLo-2), PRNCR1 (CARLo-3), PCAT2 (CARLo-4), and CASC11 (CARLo-7), have been identified [101]. Recently, the analysis of lncRNA expression in different CRC molecular phenotypes highlighted the decreased expression of UCA1 in tumors with Mismatch Repair (MMR) defaults compared to tumors without such defaults [19]. The following sections focus on the role of UCA1 to integrate research on different cancer cells in order to decipher its putative functions and mechanisms of regulation in CRC cells.

## 3. UCA1 Expression in Colorectal Cancer 

### 3.1. Association of UCA1 Transcript Expression with Colorectal Cancer

Urothelial Cancer Associated 1 (UCA1) was first discovered in bladder cancer [102] and its long transcript is also the nominated Cancer Upregulated Drug-Resistant transcript (CUDR) [103]. Actually, three UCA1 transcript isoforms of 1.4 kb, 2.2 kb, and 2.7 kb have been described, but, in general, the most abundant 1.4 kb isoform is studied [104]. Analysis of UCA1 expression and patient survival data from the TCGA dataset shows that its expression was correlated with increased hazard ratio in different types of cancers, in particular with pancreatic adenocarcinoma (Table 2, [105,106,107]). Although several patient studies reported that a high expression of UCA1 is correlated with bad disease prognosis in CRC (the number of patient of these studies N = 80 [108], N = 54 [109], N = 90 [110] and the N = 530 for the Asian meta-analysis [111]), no such evidence was observed in the TCGA COAD-READ study (Table 2). Since separated analyses of colon (COAD) and rectal (READ) adenocarcinoma patients showed different Kaplan Meier survival curves, this discrepancy may originate from mixed colon and rectal adenocarcinoma patients in several studies (e.g., known mixed COAD:READ patient groups in [108,110]). Recently, a genome-wide analysis of lncRNA expression in different CRC phenotypes was realized, highlighting the decreased expression of UCA1 in tumors with Mismatch Repair (MMR) defaults compared to tumors without such defaults [19]. These differences are in line with the notion that different primary tumor locations (COAD vs. READ) and, therefore, carcinogenesis pathways define the molecular characteristics and epigenetic signature of the tumor [112]. 

### 3.2. Regulation of UCA1 Transcript Expression

The UCA1 gene encodes 3 exons located on chromosome 19 and it is highly expressed in cancer cells. Indeed, its transcription is up-regulated by diverse oncogenic pathways. The Ras-responsive transcription factor Ets-2 was shown to regulate UCA1 transcription in both bladder and colorectal cells [109,114], UCA1 is upregulated by the major inducer of epithelial-mesenchymal transition (EMT) TGFβ in gastric and breast cancer cells [115] and by mediators of chemoresistance like Hippo (TAZ/YAP/TEAD) signaling in bladder and breast cancer cells [116,117]. BMP9 has an ambiguous role in tumor progression, but it was recently shown that BMP9 stimulated UCA1 expression in bladder cancer cells [117]. Interestingly, in these cells, UCA1 expression was also stimulated during hypoxia via Hypoxia-Inducible Factor-1α (HIF1α) and the secretion of UCA1-enriched exosomes was increased under those conditions [118,119].

Several chromatin remodeling factors inhibit UCA1 transcription. Although the transcription factor CCAAT/enhancer binding protein α (C/EBPα) upregulated the UCA1 expression [120], this activation was inhibited by the tumor repressor and part of an SWI/SNF chromatin remodeling complex, ARID1A [121]. Epigenetic inhibition of UCA1 in breast cancer cells was mediated by the Special AT-rich sequence Binding-protein 1 (SATB1) [122]. The Coactivator of AP1 and Estrogen Receptor (CAPERα)/ T-box3 (TBX3) repressor complex that mediates an arrest of cell growth also downregulated UCA1 in embryonic kidney cells [123].

Levels of UCA1 transcripts are also regulated post-transcriptionally; the RNA stability of UCA1 was downregulated by the interaction with insulin-like growth factor 2 messenger RNA binding protein (IMP1) [124] and by the interaction with miR-1 [125], whereas binding of UCA1 to heterogeneous nuclear ribonucleoprotein I (hnRNPI) increased its stability [126]. It remains to be explored if the described regulation of transcript levels in diverse cancer cells also regulates UCA1 in colorectal cells.

## 4. UCA1-Mechanism of Regulation

### 4.1. UCA1-Regulated Transcription

In common with a lot of other lncRNAs, UCA1 can regulate the transcription of genes via epigenetic modifications (Table 3). Recent studies showed that UCA1 can physically associate with EZH2 and suppress transcription via histone methylation (H3K27me3) on the promoter of cell cycle genes p21cip and p27Kip1 [43,44] and stimulate cyclin D1 expression [43]. The binding of UCA1 to transcription regulating complexes can also function as a decoy. In gallbladder cancer cells, UCA1 interacted with Brahma related gene 1 (BRG1) of the chromatin SWI/SNF remodeling complex and prevented its binding to the p21 promoter locus [52]. Binding of UCA1 to heterogeneous nuclear ribonucleoprotein I (hnRNPI) in breast cancer cells resulted in the decreased stimulation of the p27 promoter by hnRNPI [126]. Another mechanism of transcriptional regulation by UCA1 occurs in hepatocytes where the UCA1/CUDR-induced chromatin loop recruits the transcription insulator CTCF and β-catenin enhancer resulting in the upregulation of β-catenin transcription [72]. Zhang et al. performed RNA immuno-precipitation assays showing a direct interaction of UCA1 with the mediators MOB1, Lats1, and YAP of the Hippo pathway, and demonstrated a major role of UCA1 for nuclear translocation of YAP and pancreatic cancer cell migration and invasion [107]. This pathway is also important in 5FU-chemoresistance CRC [127].

### 4.2. UCA1 and miRNA-Mediated Decay 

**Figure 2:**
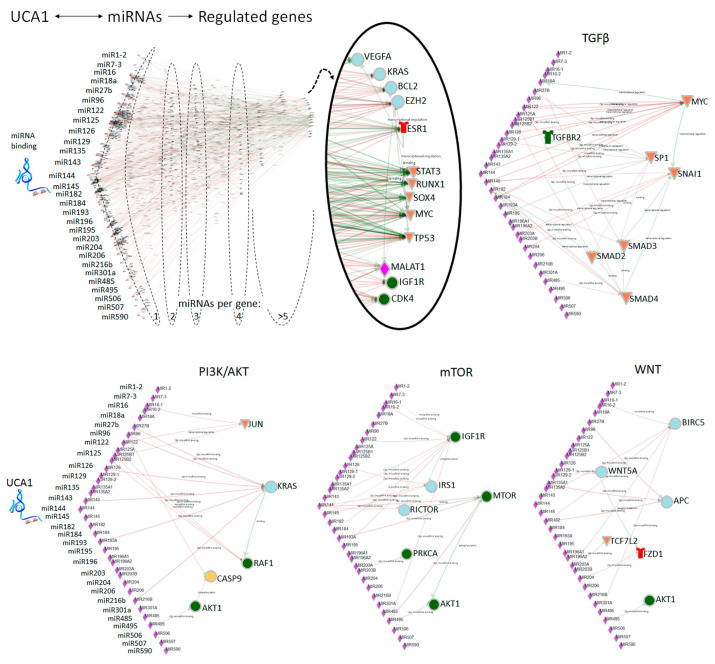
The regulation by Urothelial Cancer Associated 1 (UCA1)-associated miRNAs. All 29 miRNAs published to interact with UCA1 (Table 4) were submitted to the miRNA network analysis tool ONCO.IO and the downstream-regulated genes are visualized (https://onco.io/main.php). In the overall UCA1/miRNA image, genes were sorted based on the number of miRNAs bound per gene. The genes associated with over 5 miRNAs (circled) include VEGF, KRAS, BCL2, EZH2, receptor ESR1; transcription factors: STAT3, RUNX, SOX4, MYC, TP53; lncRNA MALAT; and the kinases IGF1R and CDK4. Genes that were associated with the indicated signaling pathways are also represented in individual images.

With the exploring of the miRNA pathways, it has become clear that lncRNAs play an important role to fine-tune miRNA-mediated decay. In the last lustrum, over 40 articles have described the indirect regulation by UCA1 through sequestering of miRNAs and interfering with the degradation of downstream gene transcripts. At least 29 miRNAs were shown to interact with UCA1 (Table 4) and overall 32 different genes were reported with an altered expression mediated by the UCA1/miRNA interaction. In addition, our analysis of putative miRNA binding with miRCore [128], showed that another 9 miRNAs not previously described, may bind to the UCA1 transcript (highlighted in Table 4). These miRNAs also play a role in CRC (Table 4; references in “CRC” column). Focusing on CRC cells, the interaction of UCA1 with miR-143, first reported in bladder cancer cells [129], was confirmed and implicated UCA1 as an upstream effector of mTOR activation and as a regulator of K-Ras expression ([130], Figure 2). Furthermore, UCA1 could sponge endogenous miR-204-5p and inhibit the degradation of its targets CREB1, BCL-2 and RAB22A indicating UCA1 promotes proliferation, inhibits apoptosis and plays a role in the acquired chemoresistance of these CRC cells [110]. Overall, gene clustering analysis of the 29 UCA1-related miRNA targets with mirPath v.3 [131] showed a significant implication in cancer signaling pathways such as TGFβ, mTOR and WNT signaling. We confirmed the UCA1/miRNA-regulated genes in these pathways with the miRNA network analysis tool ONCO.IO (Figure 2). These analyses indicate that UCA1 regulates genes that play a central role in CRC.

## 5. Role of UCA1-Mediated Regulation in Colorectal Cancer 

Reciprocal to the regulation of UCA1 transcript expression by oncogenic pathways, UCA1 may regulate oncogenic pathways. UCA1 expression has been shown to stimulate factors of the WNT signaling pathway in diverse cancer cell types [72,290,291,292,293]. In CRC, WNT signaling is correlated to 5-FU chemoresistance [294] and UCA1 is induced by 5-FU treatment [295], but no direct correlation is described for UCA1 and WNT signaling in these cells. This also holds true for UCA1 regulating mediators of AKT signaling and its downstream targets in diverse cancer cells [43,102,114,296,297]. In CRC cells UCA1 was implicated in the induction of KRAS expression through the regulation of miR143 [130]. Although no direct evidence of UCA1 regulation for the TGFβ pathway in colorectal cells was shown, UCA1 acts as a competitor RNA for several miRNAs that affect this pathway. Recently Li et al. showed that the interaction of UCA1 with miR-1 and miR203a stabilized the expression of SNAI2, mediating the effects of TGFβ signaling in breast cancer cells [115]. In addition, UCA1 was shown to stimulate the ERK-MMP9 signaling in gastric cancer cells by interacting with G protein-coupled receptor kinase 2 (GRK2), stimulating its ubiquitination and degradation [298].

### 5.1. UCA1-Mediated Regulation of the Cell Cycle

**Figure 3:**
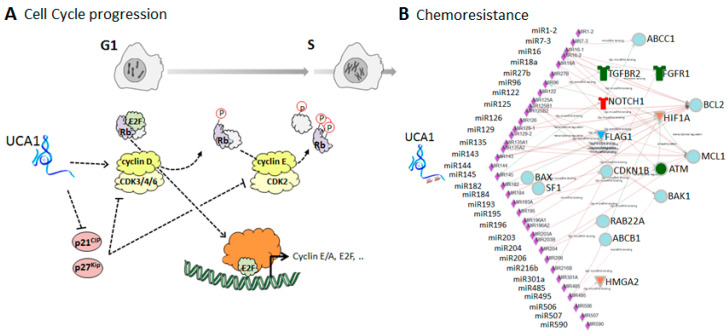
The UCA1-mediated regulation in colorectal cancer cells. (A) Schematically representation of UCA1 regulating key actors for cell cycle progression during G1 and S-phase in diverse cancer cell types. (B) All 29 miRNAs published to interact with UCA1 in diverse cancer cell types (Table 4) were submitted to the miRNA network analysis tool ONCO.IO and their interaction with chemoresistance-related genes were visualized (Receptors: TGFBR2, NOTCH1, FGFR1; Transcriptional regulating factors: HMGA2 and HIF1A; cell cycle kinase ATM, CDKN1 (p27); FLAG1, RAB22A, BAK1, BAX, ABCB1 (MDR1), ABCC1 (MRP1), MCL1, and BCL2).

UCA1 stimulates cell proliferation, and silencing its expression in cancer cells has been shown to arrest the cell cycle in the G0/G1 phase (in CRC [108,109] and other cancer cells [43,44,225,299,300,301]). Several key players in cell cycle progression are regulated by UCA1 (Figure 3A). Cell cycle progression from the G1 to S phase relies on the activation of E2F transcriptional regulation. Activation is mediated by dissociation of E2F from the Rb-complex after the phosphorylation of Rb by CDK-cyclin. From this phosphorylation complex, UCA1 increases cyclin D1 expression [43,300] and maintains CDK phosphorylation activity through the repression of its inhibitors p21CIP and p27Kip [44,52,126,130,189]. Moreover, during the G1-phase, the cyclin D1 expression and its binding to the CDK inhibitors increased, resulting in less binding of these inhibitors to cyclin E/Cdk2 complexes and acceleration of the cell cycle progression. This mechanism is further stimulated by the fact that UCA1 can upregulate cMYC, either by binding to cyclin D1 [302] or by sequestering miR-135 [162], respectively, in the liver and in thyroid cancer cells. This evidence was obtained in different types of cancer cells, but overall these studies show that UCA1 interferes at different levels with cell cycle regulation.

### 5.2. Association of UCA1 with Chemoresistance

The high expression of UCA1 is correlated with a bad cancer prognosis, which is probably related to the induction of chemotherapy drug resistance. In fact, UCA1 levels are further increased upon the development of chemoresistance to cisplatin in oral squamous cell carcinoma, bladder cancer and gastric cancer cells [148,173,189,290], to tamoxifen in breast cancer cells [147], to paclitaxel in ovarian cancer cells [160], to doxorubicin in gastric cancer cells [148], and to 5-fluorouracil in gastric cancer and CRC cells [110,148]. The effects UCA1 has on cell cycle progression and on cell proliferation is probably an important aspect of chemoresistance in these cancers. Furthermore, UCA1 affects chemoresistance by sequestering miRNAs implicated in oncogenic pathways (miR-18a, [147]; miR-27b [148]; miR-129, [160]; miR-184, [173]; miR-196a-5p [189]). In particular, in CRC cells, UCA1/miR-204-5p interaction affects the chemoresistance-related genes CREB, Bcl2, and Rab22a [110]. Chemoresistance is also modulated via miR-204 by regulation of HMGA2 in CRC cells [202] and by TGFβ-R2 in gastric cancer cells [303]. In addition, other UCA1-binding miRNAs affect these chemoresistance-related genes (Figure 3B). Although for several UCA1-binding miRNAs no direct relation between chemoresistance and UCA1 interference was studied, these miRNAs were shown to be associated to chemoresistance in CRC (miR-96 [304]; miR-129 [158]; miR-135a [305]; miR-182 [306]; miR-143, miR-145 [307]; miR-195-5p [308,309]; miR-203 [310,311,312]; miR-204-5p [110]; miR-206 [313]; miR-506 [223]). In addition, miR-27b, miR145, miR216, and miR125a-5p are related to FOLFOX resistance in CRC [314].

### 5.3. UCA1 in Colorectal Cancer Diagnosis and Therapy

Initially, UCA1 was proposed as a predictive biomarker for the prognosis and survival of CRC patients [108,109,110,111]. Since UCA1 expression in CRC may depend on primary tumor localization and molecular subtypes, its prognostic value may be restrictive to different CRC subclasses. Interestingly, a recent evaluation of tumor-derived exosomes in cancer diagnosis showed that UCA1 is not only expressed in gallbladder cancer exosomes [118], but also in exosomes isolated from the serum of CRC patients [315]. Whether the implication of UCA1 in several oncogenic pathways makes it a good target for therapy remains to be investigated. Invalidating an lncRNA, like UCA1 in CRC, may have the advantage of both inhibiting epigenetic silencing through chromatin remodeling for several tumor suppressor genes and stimulating the miRNA-mediated mRNA degradation of oncogenes due to a decreased ceRNA level. In that aspect, a recent innovation was the use of an artificial lncRNA targeting multiple miRNAs in hepatocellular carcinoma cells [316].

## 6. Conclusions

The lncRNA UCA1 has, like other lncRNAs, diverse functions and can affect both epigenetic and transcriptional gene regulation, as well as posttranscriptional regulation by acting as a ceRNA for diverse miRNAs. UCA1 has been studied in a wide range of cancer cells, including colorectal cancer. Extrapolating the role of UCA1 in different cancer cells to CRC cells suggests a role for UCA1 in cell cycle progression and cell proliferation, which is highly relevant to tumor growth. In addition, UCA1 plays a role in CRC chemoresistance, although the implicated mechanisms remain to be studied. It will also be worthwhile to identify more UCA1/EZH2-silenced target genes, to asses whether UCA1 is a driver of carcinogenesis by silencing key tumor repressor genes. UCA1 is probably not a strong general prognostic marker for CRC as its overexpression is dependent on the primary tumor site (colon vs. rectal) and molecular characteristics, such as the microsatellite stability profile. Nevertheless, studying the UCA1-regulated genes and miRNA decoy function in CRC cells may reveal novel pathways and potential new therapeutic targets for managing CRC.

## Figures and Tables

**Table 1:** The examples of long non-coding RNAs in cancer cells.

	lncRNA	References
**DNA organization**		
**Speckle formation**		
	NEAT1	[23,24,25]
	MALAT1	[25,27]
**Histone modification**		
EZH2	AFAP1-AS1	[36]
	BLACAT1	[37]
	CRNDE	[38]
	HOTAIR	[39]
	HOXA-AS2	[31]
	HULC	[40]
	PINT	[41]
	SH3PXD2A-AS1	[30]
	SNHG17	[42]
	UCA1	[43,44]
WDR5	HOTTIP	[45]
	GClnc1	[46]
	HOXD-AS	[47]
LSD1	HOTTAIR	[48]
	FOXP4-AS1	[49]
	HOXA-AS2	[31]
**SWI/SNF chromatin modulation**		
BRG1	lncTCF7	[50]
	lncFDZ6	[51]
	NEAT1	[21]
	UCA1	[52]
SNF5	SChLAP1	[34]
BAF200a	MVIH	[35]
**RNA interaction**		
**miRNA-precursors**		
miR-675	H19	[53,54]
miR-545/374a	Ftx	[55]
miR-143, -145	NCR143/145	[56]
miR-31	LOC554202	[57]
miR-125b-2, miR-99a and let-7c	MONC	[58]
miR-100, miR-125b-1 and let-7a-2	MIR100HG	[58]
Let-7c, miR99a and miR125b	LINC00478	[59]
**Protein interaction**		
**Protein stability**		
P53	PANDA	[60]
SREBP-1c	MALAT1	[61]
SLUG	SNHG15	[62]

**Table 2:** The association of Urothelial Cancer Associated 1 (UCA1) transcript expression with cancer in The Cancer Genome Atlas (TCGA) datasets.

TCGA Cancer Classification	Total Patients Number (N); N in Low vs. High Risk Group	Log Rank Equal Curves	Hazard Ratio (95% CI)	*p* Value
Acute Myeloid Leukemia	N = 149; 138 vs. 11	*p* = 0.77	1.12 (CI 0.52; 2.43)	*p* = 0.77
Bile Duct Cholangiocarcinoma	N = 35; 16 vs. 19	*p* = 0.15	2.06 (CI 0.75; 5.64)	*p* = 0.16
Bladder—Urothelial Carcinoma	N = 389; 112 vs. 277	*p* = 0.0034	1.75 (CI 1.2; 2.56)	*p* = 0.0038
Breast invasive carcinoma—July 2016	N = 962; 844 vs. 118	*p* = 0.29	1.31 (CI 0.79; 2.15)	*p* = 0.29
Cervical squamous cell carcinoma and endocervical adenocarcinoma	N = 191; 121 vs. 70	*p* = 0.051	1.82 (CI 0.99; 3.35)	*p* = 0.054
Colon and Rectum adenocarcinoma:	N = 422; 151 vs. 371	*p* = 0.66	1.1 (CI 0.72; 1.69)	*p* = 0.66
Colon	N = 350; 197 vs. 153	*p* = 0.51	0.86 (CI 0.54; 1.36)	*p* = 0.51
Rectum	N = 57; 39 vs. 18	*p* = 0.0075	4.54 (CI 1.35; 15.27)	*p* = 0.014
Esophageal carcinoma	N = 184; 148 vs. 36	*p* = 0.29	0.72 (CI 0.38; 1.33)	*p* = 0.29
Head and Neck squamous cell carcinoma	N = 506; 304 vs. 198	*p* = 0.45	1.11 (CI 0.85; 1.46)	*p* = 0.45
Kidney PAN cancer	N = 892; 715 vs. 77	*p* = 0.67	1.11 (CI 0.68; 1.83)	*p* = 0.67
Liver hepatocellular carcinoma	N = 361; 318 vs. 43	*p* = 0.025	1.68 (CI 1.06; 2.66)	*p* = 0.027
Lung adenocarcinoma	N = 475; 384 vs. 91	*p* = 0.0041	1.69 (CI 1.18;2.44)	*p* = 0.0046
Lung squamous cell carcinoma	N = 175; 123 vs. 52	*p* = 0.93	0.98 (CI 0.61; 1.58)	*p* = 0.93
Ovarian serous cystadenocarcinoma	N = 247; 25 vs. 222	*p* = 0.21	0.72 (CI 0.43; 1.21)	*p* = 0.21
Pancreatic adenocarcinoma	N = 176; 154 vs. 22	*p* = 1.766 × 10^-0.5^	2.94 (CI 1.75; 4.92)	*p* = 4.249 × 10^-0.5^
Stomach and Esophagous adenocarcinoma	N = 440; 220 vs. 220	*p* = 0.90	0.98 (CI 0.72; 1.33)	*p* = 0.90
Stomach adenocarcinoma	N = 352; 135 vs. 217	*p* = 0.70	1.07 (CI 0.75; 1.52)	*p* = 0.70
Testicular Germ Cell Tumors	N = 133; 105 vs. 28	*p* = 0.19	3.39 (CI 0.48; 24.1)	*p* = 0.22
Uterine Corpus Endometrial Carcinoma	N = 247; 130 vs. 117	*p* = 0.084	1.85 (CI 0.91; 3.75)	*p* = 0.089
Kaplan-Meier survival curve statistics are reported from the TCGA cohort data using the SurvExpress portal [113].				

**Table 3:** The interaction of UCA1 with transcription regulating complexes.

Interaction	Complex	Target	Cells	Ref.
Enhancer of zeste homolog 2 (EZH2)	polycomb repressive complex-2	Cycline D1	gastric cancer	[43]
		p27Kip1	hepatocarcinoma	[44]
CCCTC-binding factor (CTCF)	chromatin looping with RNA polII and P300	HULC	Embryonic hepatocyte-like	[72]
Brahma related gene 1 (BRG1)	SWI/SNF chromatin remodeling	p21	bladder cancer	[52]
MOB1, Lats1, and YAP	YAP-TEAD transcription complex	Hippo pathway	Pancreatic cancer	[107]

**Table 4:** The UCA1-mediated regulation of miRNA targets.

UCA1-Mediated miR Regulation (Sponges/Competing Endogenous RNA)					miR-Mediated Regulation	
miRNA *	miR-Target	Type of Cells		Targets **	Biological Process ***	CRC
**miR-1**	Hes1	neural stem cell	[132]	915		
		bladder cancer cells	[125]			
	Slug	Breast cancer	[115]			
**miR-7**	EGFR	gastric cancer	[133]	884	transmembrane receptor protein tyrosine kinase signaling pathway	[134,135,136,137,138,139,140,141]
miR-16	MDR1	chronic myeloid leukemia	[142]	1646	protein folding, rRNA metabolic process, tRNA aminoacylation for protein translation, protein acetylation, regulation of sequence-specific DNA binding transcription factor activity, nuclear transport, nucleobase-containing compound transport, tRNA metabolic process, RNA localization, protein targeting, cellular component biogenesis	[143]
	GLS2	bladder cancer	[144]			
**miR-18a**	YAP	breast cancer	[145]	N.A.	N.A.	[146]
	HIF1α	breast cancer	[147]			
miR-27b		gastric cancer	[148]	447	regulation of cell cycle, intracellular protein transport	[149]
**miR-122**		breast cancer	[124]	580	N.S.	[150]
**miR-125**	HK2	acute myeloid leukemia	[151]	899		
miR-126	RAC1	human myelogenous leukemia	[152]	152	N.S.	-
**miR-129**	SOX4	renal cell carcinoma	[153]	499	N.S.	[154,155,156,157,158,159]
	ABCB1	ovarian cancer	[160]			
**miR-135a**		pancreatic cancer	[106]	121	N.S.	[161]
	cMYC	thyroid cancer	[162]			
**miR-143**	mTOR (cyclin D1, p27)	**colorectal cancer**	[130]	478	N.S.	[130,163]
	ERBB3 BCL-2	breast cancer	[164]			
	FOXO1	cardiomyocyte	[165]			
	HMGB1	bladder cancer	[166]			
	HK2	bladder cancer	[129]			
miR-145	FSCN1 ZEB1/2	bladder cancer	[167]	263	cell proliferation, cytokinesis, negative regulation of apoptotic process, anatomical structure morphogenesis, regulation of cell cycle, MAPK cascade	[168,169]
**miR-182**	p53 (iASPP)	Glioma	[170]	189	N.S.	[171]
	PFKFB2	glioblastoma-associated stromal cells	[172]			
**miR-184**	SF1	oral squamous cell carcinoma	[173]	29	N.S.	[174]
**miR-193a**	HMGB1	lung cancer	[175]	144	N.S.	[176,177,178,179,180]
	ERBB4	non-small cell lung cancer	[181]			
miR-195	ARL2	bladder Cancer	[182]	692	angiogenesis, cell proliferation, cytokinesis, anatomical structure morphogenesis, mitosis, regulation of transcription from RNA polymerase II promoter	[183,184,185,186,187,188]
miR-196a	CREB	bladder cancer	[189]	450	RNA splicing, via transesterification reactions, response to stress, organelle organization	[190,191,192,193]
**miR-203**	Snail2	hepatocellular carcinoma	[194]	528	N.S.	[195,196,197,198,199,200]
	Slug	Breast cancer	[115]			
miR-204	CREB1, BCL2, RAB22A	**colorectal cancer**	[110]	488	N.S.	[110,201,202,203]
	MMP-13	chondrocytes	[204]			
	Sirt1	prostate cancer	[205]			
	Sox4	esophageal cancer	[206]			
	BRD4	thyroid cancer	[207]			
miR-206	VEGF	cervical cancer	[208]	95	pentose-phosphate shunt, chromatin assembly, chromatin remodeling, negative regulation of apoptotic process, chromatin organization, regulation of phosphate, metabolic process	-
miR-216b	FGFR1	hepatocellular carcinoma	[209]	235	protein targeting	[209,210,211,212,213]
miR-485	MMP14	epithelial ovarian	[214]	505	N.S.	-
miR-495	p21	renal cell carcinoma	[215]	241	N.S.	[216,217]
miR-506	COTL1	non-small cell lung cancer	[218]	180	N.S.	[219,220,221,222,223,224]
miR-507	FOXM1	melanoma	[225]	169	N.S.	-
miR-590	CREB	gastric cancer	[226]	419	N.S.	[227,228,229,230]
**miR-22**	-	-	-	221	negative regulation of apoptotic process, regulation of transcription from RNA polymerase II promoter	[231,232,233,234,235,236,237,238,239,240,241]
**miR-23a**	-	-	-	353	N.S.	[242,243,244,245,246,247]
**miR-26a**	-	-	-	531	Mitosis, regulation of cell cycle, phosphate-containing compound, metabolic process	[248,249,250,251,252,253,254,255,256]
**miR-103a/107/107ab**	-	-	-	857	cytoskeleton organization, cell cycle	[257,258,259]
**miR-124**	-	-	-	1520	regulation of binding, cytokinesis, transmembrane receptor, protein tyrosine kinase signaling pathway, regulation of cell cycle	[219,255,260,261,262,263,264,265,266,267,268,269,270,271,272,273,274]
**miR-138**	-	-	-	239	N.S.	[275,276,277,278]
**miR-190**	-	-	-	770	RNA splicing, via transesterification reactions	-
**miR-214**	-	-	-	352	N.S.	[279,280,281,282,283]
**miR-455**	-	-	-	557	N.S.	[284,285,286,287]

* in bold = miRNA target site predicted at UCA1 gene by miRcode [128]; ** = number of miRNA-validated targets (identified in ChemiRs/mirTAR database for 3p and 5p mature miRNA [288]) *** = Panther Go-Slim biological processes that present a ≥2-fold enrichment for miRNA-validated targets (PANTHER [289]; Overrepresentation Test (version 20171205/version 13.1)). N.A. = miRNA data not available in ChemiRs database. N.S. = No statistically significant results (FDR > 0.05). “CRC”-column: references of reports implicating miRNAs in CRC.
