# Correction: Neve et al. Epigenetic Regulation by lncRNAs: An Overview Focused on UCA1 in Colorectal Cancer. *Cancers* 2018, *10*, 440

**DOI:** 10.3390/cancers17060914

**Published:** 2025-03-07

**Authors:** Bernadette Neve, Nicolas Jonckheere, Audrey Vincent, Isabelle Van Seuningen

**Affiliations:** Inserm UMR-S 1172, Centre de Recherche Jean-Pierre AUBERT Neurosciences et Cancer (JPArc), Team “Mucins, Epithelial Differentiation and Carcinogenesis”; University Lille; CHU Lille, 59045, Lille CEDEX, France; nicolas.jonckheere@inserm.fr (N.J.); audrey.vincent@inserm.fr (A.V.); isabelle.vanseuningen@inserm.fr (I.V.S.)

## Text Correction

There was an error in the original publication [1], a correction has been made to Section 2.1.1, Paragraph 2, sixth sentence:

Interestingly, the tumor suppressor KLF2 gene is reported to be silenced in CRC cells by several lncRNAs through this mechanism (e.g., SH3PXD2A-AS1, HOXA-AS2 [30,31]), whereas for most genes, only a unique lncRNA/EZH2 combination is known.

## Update to Citations

Since the original publication of this review [1] in 2018, several cited papers concerning the UCA1-mediated regulation of miRNAs have been found to contain dubious figures, been identified as papermill-generated, or been retracted (https://pubpeer.com/). In order not to spread invalid citations and to allow our review to remain as a point of reference, we have decided to update our review by removing 32 dubious references and five citations that are no longer relevant (out of 353, original references [30,38,45,49,54,67,121,134,141,150,160–163,166,182,183,185,187,192,195,209,226,237–240,245,251,267,275,290,295,298,311,312,322] are removed). These references were cited in Tables 3 and 4 as examples amongst other miRNAs papers and therefore do not change the overall conclusion of the review.

The reference in Section 4.1. concerning another example of UCA1 action (on the telomeric repeat binding factor 2 (TRF2) promoter region) has also been retracted, and this part was removed from the review. The overall conclusion of the review remains valid, although novel references (from 2018 onward) are not included in the updated version. This correction was approved by the Academic Editor. The original publication has also been updated.
